# Split phenomena in amyotrophic lateral sclerosis: Current evidences, pathogenetic hypotheses and diagnostic implications

**DOI:** 10.3389/fnins.2022.1100040

**Published:** 2023-01-09

**Authors:** Stefano Zoccolella, Alessia Giugno, Giancarlo Logroscino

**Affiliations:** ^1^Center for Neurodegenerative Diseases and the Aging Brain, University of Bari Aldo Moro at Pia Fondazione “Card. G. Panico”, Tricase, Italy; ^2^Neurology Unit, Azienda Sanitaria Locale (ASL) Bari, San Paolo Hospital, Bari, Italy; ^3^Department of Medical Sciences, Institute of Neurology, Magna Græcia University, Catanzaro, Italy; ^4^Department of Translational Biomedicine and Neuroscience (DiBraiN), University of Bari Aldo Moro, Bari, Italy

**Keywords:** amyotrophic lateral sclerosis, motor neuron, split-hand sign, split-leg sign, neurophysiology, split-hand index, split-leg index, split-elbow index

## Abstract

Amyotrophic lateral sclerosis (ALS) is the most common motor neuron disease and has emerged among the disorders with the largest increasing incidence in Western countries. Although the diagnosis is based on clinical grounds, electromyography (EMG), and nerve conduction studies (NCS) play a crucial role to exclude other potential etiologies of lower motor neuron (LMN) dysfunction. Based on clinical grounds, a peculiar pattern of dissociated atrophy of the intrinsic hand and foot muscles, termed the “split-hand” (SH) and “split-leg” (SL) signs, has been described in a significant proportion of subjects with ALS, even at the early stages of the disease, when symptoms are focal. These signs are rare in neurological and non-neurological diseases other than ALS. In this review, we discussed current evidences concerning SH and SL signs, their pathogenetic hypotheses and neurophysiological findings. We also analyze whether SH and SL signs can be reliable markers in the differential diagnosis and in the prognosis of ALS.

## 1. Introduction

**FIGURE 1:**
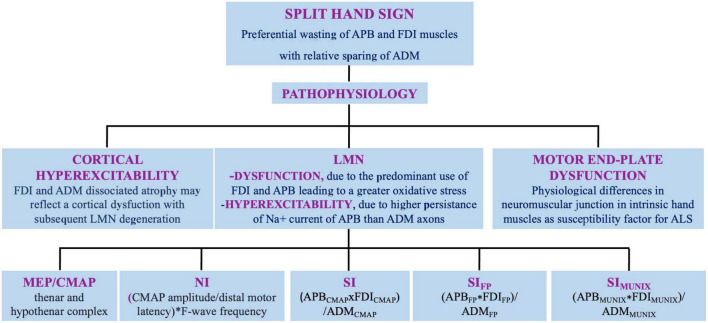
Split hand sign in ALS: definition, pathophysiology, and neurophysiologic indexes. APB, abductor pollicis brevis muscle; FDI, first dorsal interosseus; ADM, abductor digiti minimi muscle; LMN, lower motor neuron; MEP, motor evoked potential; CMAP, compound muscle action potential; NI, neurophysiological index; SI, split hand index; SIFP, SI derived from F-waves; SIMUNIX, SI based on MUNIX software.

**FIGURE 2:**
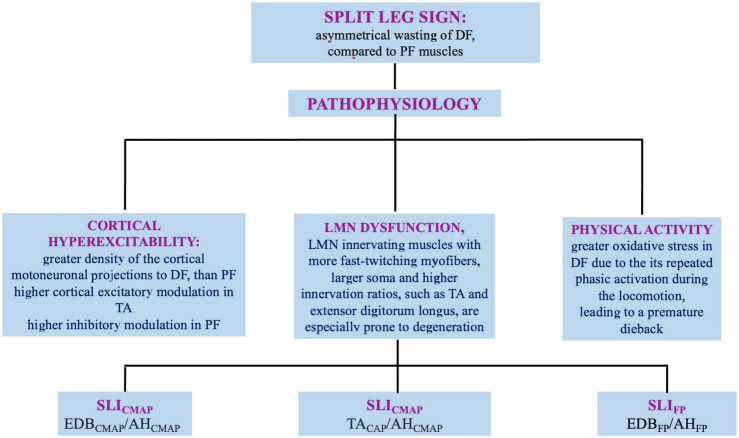
Split leg sign in ALS: definition, pathophysiology, and neurophysiologic indexes. DF, dorsal flexors; PF, plantar flexors; LMN, lower motor neuron; SLI, split leg sign; CMAP, compound muscle action potential; EDB, extensor digitorum brevis; AH, abductor hallucis; TA, tibialis antherior; SLIFP, SLI derived from F-waves.

Amyotrophic lateral sclerosis (ALS) involves upper (UMN) and lower motor neuron (LMN), and is fatal on average within 4 years from onset. The disease onset is often focal, embroiling face or distal segments of arms/legs and steadily advances over time and space (Kiernan et al., 2011). Although the diagnosis is based on clinical grounds, electromyography (EMG) and nerve conduction studies (NCS) help either to rule out LMN dysfunction of different etiologies and to identify LMN signs in muscles not clinically involved (Shefner et al., 2020). Several clinical and neurophysiological studies reported a dissociated pattern of muscle wasting and hypotrophy of intrinsic hand, elbow, and foot muscles in ALS, named respectively, “split-hand” (SH), split-elbow (SE), and “split-leg” (SL) signs (Corcia et al., 2021). In this review, we discuss current evidences, pathogenetic hypotheses and neurophysiological indicators of SH, SE, and SL (Figures 1, 2).

## 2. Split-hand sign

The preferential wasting of abductor pollicis brevis (APB) and first dorsal interosseous (FDI), with relative sparing of the abductor digiti minimi (ADM) in ALS was first described in 1994 (Wilbourn and Sweeney, 1994). Further studies defined this phenomenon as the “SH” (Corcia et al., 2021). Independently of the site of onset, approximately 70% of patients with ALS presents SH at diagnosis and almost all cases during the course of the disease (Hu et al., 2021). These findings emphasized a potential role for SH as diagnostic indicator of ALS (Corcia et al., 2021). However, the specificity of SH still remains debated as SH has been reported in other conditions as spinal muscular atrophy, Kennedy’s disease, spinocerebellar ataxia-3 and post-polio syndrome (Schelhaas et al., 2003; Shibuya et al., 2020; Corcia et al., 2021). Moreover, a physiological non-progressive, age-related preferential atrophy of APB and FDI has been described and defined the “senile amyotrophy” (Op de coul, 1970).

### 2.1. Pathophysiology

The dissociated atrophy of hands muscles does not reflect nerve trunks or root territories. FDI and ADM muscles are both innervated by the ulnar nerve arising from C8–T1 nerve roots. The median nerve innervates APB, and also arises from C8–T1 roots (Corcia et al., 2021). Therefore, four pathophysiologic mechanisms of SH have been proposed (Figure 1).

#### 2.1.1. Corticomotoneuronal dysfunction

The motor cortex may be primarily involved in SH, as although FDI and ADM are innervated by the same myotomes, they depict a dissociated atrophy, that can reflect a cortical mechanism (Weber et al., 2000; Menon et al., 2013b). The intrinsic lateral hand muscles are involved in precision movements that depend on normal corticospinal-tract function (Menon et al., 2013b; Xu and Fan, 2013). APB or FDI cortical motor neurons far outnumber those of ADM (Kuwabara et al., 1999) and their corticospinal connections are more extensive than those of ADM, resulting in greater susceptibility to glutamate excitotoxicity of APB and FDI LMN. The smaller cortical motor evoked potentials (MEPs) obtained from the thenar muscles compared to ADM may indicate a “shrinkage” of cortical representation of the former (Weber et al., 2000). Based on these observations, the dysfunction of corticomotoneuronal network may represent the determinant key of dissociated hand wasting (Weber et al., 2000; Corcia et al., 2021). Other neurophysiological studies, however, argued against this hypothesis, finding no significant difference between FDI and ADM in the cutaneous silent period elicited stimulating a digital sensory nerve in patients with ALS (Cengiz et al., 2018).

#### 2.1.2. LMN dysfunction

SH may reflect the predominant use of thumb and index fingers, that leads to a greater oxidative stress or metabolic demand on APB and FDI LMN (Weber et al., 2000; Corcia et al., 2021). The hypothesized greater resistance to degeneration of ADM LMN may be related to a greater reinnervation capacity of its axons (Murray and Jankelowitz, 2011; de Carvalho and Swash, 2019; Corcia et al., 2021).

#### 2.1.3. LMN hyperexcitability

Experimental studies on peripheral axonal excitability evidenced that in normal subjects the Na^+^ current of APB motor-axons are more persistent than those of ADM. Consistently, case-control studies demonstrated that the hyperexcitability in ALS is predominant in APB, compared to ADM axons (Shibuya et al., 2013, 2020). However, in other studies excitability changes did not follow a SH pattern, as the increase in axons excitability resulted similar in APB and ADM, arguing against a peripheral contribution (Menon et al., 2013b; Menon and Vucic, 2014).

#### 2.1.4. Motor-end plate dysfunction

A single repetitive nerve stimulation study was conducted in patients with ALS with normal strength hand, normal median, and ulnar compound muscle action potentials (CMAPs) and in normal subjects. In patients with ALS the CMAPs decrement resulted higher in ABD and FDI compared to ADM, whereas the decremental pattern resulted similar in all muscles in controls. Authors hypothesized that differences in neuromuscular junctions in intrinsic hand muscles mirroring SH sign can be a susceptibility factor for ALS (de Carvalho and Swash, 2019).

### 2.2. Neurophysiology

Several neurophysiological methods have been used through the years to quantify the SH (Figure 1 and Table 1). The first indicator was a cortical/peripheral ratio of the thenar and hypothenar muscles, obtained by peripheral nerve and transcranial magnetic stimulations (TMSs). Compared to controls, the ratio resulted significantly lower in the thenar but not in the hypothenar complex in ALS (Weber et al., 2000). A further approach proposed a “neurophysiological index” (NI) from ulnar nerve:


$$NI=\left(\begin{array}{c} \frac{CMAPamplitude}{distal}\\ motor\,latency \end{array}\right)*Fwave\,frequency$$

**TABLE 1:** Neurophysiological index (NI) of split-hand (SH) and split-leg signs (SLS).

References	Country	Study design	Number of subjects	Neurophysiological assessment	Results
Kuwabara et al., 1999	Japan	Case-control study	40	-CMAPs recorded from APB and ADM. -MUNE of the APB and ADM determined by dividing the maximal CMAP amplitude by a mean single MUAP. -Relative severity between the APB and ADM expressed as the MUNE ratio (APB/ADM) or CMAP ratio (CMAP amplitude of the APB/ADM).	-Smaller MUNEs in ALS patients than normal subjects in both muscles. -Significantly greater extent of motor unit loss in the APB than ADM. -Significantly smaller APB/ADM ratios in ALS than normal subjects and patients with CSA, bulbospinal muscular atrophy, or peripheral neuropathy.
Weber et al., 2000	Canada	Case-control study	29	-Cortical/peripheral ratios of MEPs/CMAPs calculated with transcranial magnetic and peripheral nerve stimulation of the thenar and hypothenar complexes.	-Significant reduction of the ratios for the thenar complex (*p* = 0.02), but not for the hypothenar, in patients with ALS.
de Carvalho and Swash, 2000	Portugal	Case-control study	105	Motor latency, conduction time, *F*-wave frequency in ulnar nerves. From the ulnar nerve studies the following NI has been identified: (CMAP amplitude/DML) × *F* frequency.	-Strong correlation between ADM CMAP reduction and weakness (*r* = 0.74, *P* < 0.001). -DML, proximal conduction time, and *F*-wave frequency were abnormal with minimally detectable weakness. In weaker ADM muscles, conduction velocities, and *F*-wave latencies were also abnormal. -Strong correlation of “ALS NI,” from the ulnar nerve studies as (CMAP amplitude/DML) × *F* frequency, with ADM weakness (*r* = 0.74, *P* < 0.001).
Schelhaas et al., 2003	The Netherlands	Consecutive patients	13	-CMAP of the thenar and hypothenar complexes.	-The study supports the theory of an intrinsic vulnerability of spinal motor neurons subserving the thenar complex.
Murray and Jankelowitz, 2011	Australia	Prospective study	20	-CMAP recorded from APB and ADM. -Nerve excitability study assessed with a computerized protocol.	Significant difference in median nerve to APB and ulnar nerve to ADM studies at the wrist differ in strength-duration time constant (*p* = 0.01), stimulus threshold and threshold electrotonus (*p* < 0.01). No significant differences concerning the recovery cycle superexcitability.
Xu and Fan, 2013	China	Retrospective study	138	-NCS of musculocutaneous, axillary, median, ulnar, radial, tibial, peroneal, and sural nerves. -EMG of bulbar, cervical, thoracic, and lumbosacral regions.	-Higher amplitude of MUAP of deltoid muscle and biceps muscle in individuals with flail arm syndromes vs. ALS (*p* < 0.05). -Higher amplitude of MUAP of first dorsal interosseous (FDI), deltoid, and biceps muscles in flail arm syndromes vs. upper brachial plexus neuropathy (*p* < 0.05).
Menon et al., 2013b	Australia	Prospective case-control study	170	-SI as (APB_CMAP_*, FDI_CMAP_)/ADM_CMAP_; -NI: (CMAP amplitude*F wave frequency %)/ DML.	-Reduced SI in patients with ALS, compared to other neuromuscular disorders, particularly in limb-onset ALS.
Menon et al., 2013a	Australia		103	-MSI as MRC score of APB divided by FPL MRC score.	-Significantly reduced MSI score in ALS compared to non-ALS patients (*p* < 0.01).
Bae et al., 2014	Korea, Australia	Prospective study	17	-SHPI = APB_CMAP_/FPL_CMAP_ -Cortical excitability assessed through transcranial magnetic stimulation (TMS).	-Significantly reduced split-hand plus index (SHPI) in ALS patients vs. controls (*p* < 0.001) in both limb- and bulbar-onset ALS. -Significantly increased MEP amplitude recorded over APB in ALS vs. controls (*p* < 0.05); -No significant differences in MEP amplitude from FPL between ALS and controls (*p* = 0.11). -Significant reduction of cortical silent period duration from thenar muscles (*p* < 0.01). Inverse correlation of APB MEP amplitude with the CMAP (*p* < 0.01) and SHPI (*p* < 0.01).
Menon and Vucic, 2014	Australia	Prospective case-control study	165	-SI correlated with total and thenar eminence MRC score; functional score obtained at ALSFRS-R score; disease progression (48-ALSFRS-R)/duration of symptoms); site of disease onset (bulbar or limb).	-Significant reduction of APB CMAP (*p* < 0.001), FDI (*p* < 0.001), and ADM (*p* < 0.001) in subjects with ALS, compared with subjects with neuromuscular disorders. -In patients with ALS: Predominant reduction in CMAP amplitudes from the APB vs. ADM (*p* < 0.001) and from the FDI vs. ADM (*p* < 0.05) muscles; Significant reduction both in subjects with definite and possible ALS. Significant correlation with total MRC score (rho = 0.7, *p* < 0.001) and MRC score from the thenar eminence (rho = 0.8, *p* < 0.001). Significant correlation with the rate of disease progression (rho = −0.4, *p* < 0.05).
Kim et al., 2015	Republic of Korea	Retrospective case-control study	138	-ADM/APB CMAP ratio. -Relationship between ADM/APB CMAP ratio and disease duration.	-The ADM/APB CMAP ratio was significantly higher in subjects with upper limbs onset-ALS than in other variants.
Simon et al., 2015	Australia, Korea, UK	Prospective case-control study	74	-MUNE, CMAP_DF_, and CMAP_PF_ -SLI = CMAP_DF_/CMAP_PF_	-CMAP_DF_ (*p* < 0.001) and CMAP_PF_ (*p* < 0.001) were significantly reduced in the affected limbs of patients with ALS vs. controls. -In patients with ALS: Significant reduction of MUNE and CMAP amplitude from PFs compared to dorsiflexors (*p* < 0.001). Correlation of SLI with strength of PF muscles (*R* = −0.56, *p* < 0.001). Greatest mean SLI in lower limb-onset ALS.
Kim et al., 2016	Republic of Korea	Case-control study	79	SI vs. SI_MUNIX_ performed on APB, FDI, ADM muscles	Significantly lower CMAP and MUNIX, SI_CMAP_, and SI_MUNIX_ in patients with ALS vs. controls. Good diagnostic accuracy for both indices, but better performance of SI_MUNIX_ than with significant difference in ROC curve than SI_CMAP_ (*P* < 0.05).
Hu et al., 2019	China, USA	Case-control study	585	-SLI = CMAP_DF_/CMAP_PF_	SLI is lower in subjects with ALS (*p* < 0.001). A SLI cutoff of 0.52 and 0.33 helped to differentiate ALS from lumbar spondylosis disease and peripheral neuropathy.
Khalaf et al., 2019	UK	Retrospective study	411	MRC score at BB vs. MRC score at TB muscle.	BB MRC score was smaller than triceps in 258 limbs, with no difference in the remaining 477 (*p* < 0.001).
Wang et al., 2019	China	Case-control study	75	-CMAPs and *F*-waves recorded over the EDB and AH muscles bilaterally. -Electrophysiological parameters for *F*-waves: latencies, chronodispersion, persistence, and mean *F*-wave amplitude. mean and maximum F/M amplitude ratios, index Freps. -Compare changes in EDB and AH CMAP amplitudes and *F*-wave variables, a ratio of the EDB and AH parameters has been applied as EDB/AH. -The variation of difference in Freps of the EBD and AH was calculated applying the formula: EDB-AH index Freps.	Subjects with lower limbs involvement vs. healthy controls depicted: Significant reduction of EDB/AH CMAP amplitude ratio in the affected legs (0.33 ± 0.21, *P* = 0.007). Significant increasing of the EDB/AH ratios for the *F*-wave latencies, mean *F*-wave amplitude, mean F/M amplitude ratio, and index Freps of the EDB-AH in the affected leg. Significant reduction of EDB/AH ratio for *F*-wave persistence.
Zheng et al., 2019	China	Case-control study	120	SI_MUNIX_ performed on APB, ADM, FDI in ALS vs. CSA.	Significant difference in ADM/APB ratio and in SI between patients with ALS vs. patients with CSA patients (*P* < 0.05). Better differential diagnostic marker resulted SLI identified by MUNIX, even in their early stages of ALS.
Shibuya et al., 2020	Japan	Case-control study	338	-SI -Multiple nerve excitability measurements.	The subjects with ALS and spinal and bulbar muscular atrophy share increased motor axonal excitability.
Xu et al., 2020	Republic of Korea	Case-control study	79	Comparison of SI as CMAP with SI from the MUNIX (SI_MUNIX_) performed on APB, FDI, and ADM muscles.	Better performance (*p* < 0.05) of SI_MUNIX_ compared with SI from CMAP.
Wang et al., 2020	China	Prospective case-control study	309	-CMAPs and *F*-waves from the APB, FDI, ADM. -SI_FP_ and SI_F/M_ as: SI = (APB*FDI)/ADM -The sensitivity and specificity of SI_FP_ and SI_F/M_ in differentiating ALS from non-ALS conditions.	-Significant reduction of SI_FP_ and increasing of SI_F/M_ in ALS patients than non-ALS (*p* < 0.001). -SI_FP_ cut off of 73.3 displayed significantly higher sensitivity and specificity (*p* < 0.001) than SI_F/M_ and SI_CMAP_ for ALS diagnosis.
Min et al., 2020	Republic of Korea	Retrospective study	232	SLI defined as EDB: CMAP_EDB_/CMAP_AH_ vs. as TA: CMAP_TA_/CMAP_AH_	-SLI at EDB was significantly reduced in ALS (*p* < 0.0001) and progressive muscle atrophy (*p* < 0.0001) than in healthy controls. -SLI at EDB was reduced in lower motor neuron (LMN) signs and increased SLI at TA in upper motor neuron (UMN) signs.
Higashihara et al., 2020	Australia	Prospective study	15	-CMAP amplitude, MUNIX, and MScan from APB, FDI, and ADM muscles on three different occasions.	-High amplitudes reproducibility of CMAP (intraclass correlation coefficients of 0.86 for APB, 0.90 for FDI, 0.96 for ADM), of MUNIX and MScan across the three muscles. -No significant correlations between MUNIX and MScan coefficients of variation and CMAP.
Liu et al., 2021	China	Prospective study	618	Split phenomena investigated evaluating muscle strength in upper and lower limbs through MRC scoring system	-Split phenomena in 22.3% antagonistic muscles for flexion and extension of the elbow, 11.9% for the wrist, 23.9% for fingers, 18.2% for the ankle, and 14.7% for toes. -The presence of muscle wasting was more common when the muscle strength was stronger than a modified MRC grade 6, regardless age at onset, gender, disease duration, region of onset, damage of pyramidal tract.
Barp et al., 2021	Italy	Cross-sectional study	82	-Nerve conduction study to analyze NI, SI, and SLI. -Significant correlation emerged between both SI and NI with deltoid and APB at upper limb.	-Associations between neurophysiological indices and muscle strength at MRC. -Associations between neurophysiological indices and both functional and respiratory status at ALSFRS-R and forced vital capacity, respectively. -Associations between neurophysiological indices and staging at Milano–Torino and King’s staging systems. -Associations between neurophysiological indices and disease progression rate. -Due to the significant correlation with progression of disease (*p* < 0.0001), “split-hand prognostic index” can be considered as a prognostic marker.
Pavey et al., 2021	Australia, Japan	Prospective study	66	Nerve conduction study to analyze SEI	SEI significantly more common (*p* < 0.05) and lower (*P* < 0.01) in ALS patients. The SEI cut-off value was of 0.62 (sensitivity = 71%; specificity = 61%).
Pechirra et al., 2022	Portugal	Cross-sectional study	244	Correlation of APB, FDI, and ADM CMAPs, and SI with age.	Significant correlation of APB, ADM, FDI CMAPs, and SI with age (*p* < 0 .001).
Lu et al., 2022	Taiwan, United States	Meta-analysis	158	SI obtained as (APB*FDI)/(ADM). The Bayesian analysis was applied for validation.	A cut-off of 7.4 for SI can identify the earlier diagnosis of ALS.

CMAP, compound muscle amplitude potentials; APB, abductor pollicis brevis muscle; ADM, abductor digiti minimi muscle; MUNE, motor unit number; MUAP, single motor unit potential amplitude; MUNE ratio, motor unit number of the APB/ADM; ALS, amyotrophic lateral sclerosis; CSA, cervical spondylotic amyotrophy; MEPs, motor evoked potentials; NI, neurological index; DML, distal motor latency; MRC, Medical Research Council; NCS, nerve conduction study; EMG, electromyogram; SI, split-hand index; MSI, muscle strength index; FPL, flexor pollicis longus; ALSFRS-R, revised-ALS functional rating scale; MUNE, motor unit number estimates; MUNIX, motor-unit number index; ROC, receiver operating characteristic; SI_MUNIX_, SI calculated from the MUNIX; SI_FP_, SI recorded from F-waves persistence; MScan, motor unit number estimation; SLI, split-leg index; DF, ankle dorsiflexion muscles; PF, plantar flexor muscles; CMAP_DF_, CMAP recorded over DF; CMAP_PF_, CMAP recorded over PF; EDB, extensor digitorum brevis; AH, abductor hallucis; index Freps, persistence of total repeater F-wave shapes; TA, tibialis anterior; BB, biceps brachii; TB, triceps brachii; SES, split-elbow sign; SEI, split-elbow index; SICI, short-interval intracortical inhibition.

Neurophysiological index resulted reproducible, sensitive to change, and correlated with ADM weakness, with a sensitivity higher than clinical measures in detecting LMN loss in asymptomatic limbs. NI had a sensitivity at least similar to revised-ALS functional rating scale (ALSFRS-R) in differentiating rapid from slowly progressive ALS (de Carvalho and Swash, 2000, 2019; Swash and de Carvalho, 2004). Another study used a NI deriving from APB, with the same formula with similar results (Xu and Fan, 2013). Several other ratios have been further proposed, using CMAP amplitudes of APB or FDI and ADM in various combinations. The most widely adopted were,


$$NI=\frac{APB}{ADM}$$

$$NI=\frac{FDI}{ADM}$$

With cut-off values: 0.6 and 0.9, respectively. These indexes resulted useful to differentiate ALS from mimic disorders in some studies (Kuwabara et al., 1999; Menon et al., 2013b; Menon and Vucic, 2014, Corcia et al., 2021), while others found low sensitivity values (Table 1, Wang et al., 2020). Thereafter, a split-hand index (SI) was developed to improve the diagnostic accuracy of SH in ALS (Menon et al., 2013b). The SI is calculated applying the following formula (Menon et al., 2013b; Menon and Vucic, 2014):


$$SI_{CMAP}=\frac{\left(APB_{CMAP}*FDI_{CMAP}\right)}{ADM_{CMAP}}$$

The diagnostic accuracy of SI has been confirmed in a large study comparing ALS to mimicking syndromes, with a cut-off value of < 5.2 demonstrating sensitivity of 74% and specificity of 80%, (Menon et al., 2013b). Further studies gave similar results, but using heterogeneous cut-off values (from <5.2 to >10, Table 1) (Kuwabara et al., 1999; Corcia et al., 2021). A recent meta-analysis of available studies identified a cut-off of < 7.4 in patients with a disease duration of 8–20 months (Lu et al., 2022). SI resulted significantly reduced in more than 60% of patients with ALS who did not meet Awaji diagnostic criteria, suggesting a diagnostic role of SI in the earliest stage (Schelhaas et al., 2003; Menon et al., 2013b; Menon and Vucic, 2014). Both NI and SI weakly correlated with strength scores of the corresponding muscles, probably due to the low sensitivity of the manual strength tests, as subjects with reduction of CMAP > 50% still display normal strength (Menon et al., 2013b; Menon and Vucic, 2014). NI and SI significantly correlated with measures of functional status (Menon et al., 2013b; Corcia et al., 2021). The combination of SI and disease duration represented the best predictor of the disease progression. The resulting SI prognostic index significantly correlated with progression and survival in a cohort of patients with ALS (Xu and Fan, 2013; Barp et al., 2021). An adapted version of SI was recently obtained from *F*-waves persistence (SI_FP_) (Wang et al., 2020):


$$SI_{FP}=\frac{\left(APB_{FP}*FDI_{FP}\right)}{ADM_{FP}}$$

SI_FP_ resulted a sensitive diagnostic neurophysiological indicator. SI_FP_ (cut-off value: 73.3) improved diagnostic performances compared to SI_CMAP_, even in the early stage of the disease (sensitivity of 86% vs. 76%) (Wang et al., 2020). However, the need to deliver 100 supramaximal stimuli for each muscle limits its usefulness in clinical practice. Another non-invasive approach to quantify the SH phenomenon is the motor-unit number index (MUNIX) (Higashihara et al., 2020). MUNIX is a simple commercialized software, based on a standardized technique to quantify MUN. MUNIX value is derived from the CMAP area and surface EMG interference pattern. A MUNIX based index (SI_MUNIX_) obtained as (Higashihara et al., 2020):


$$SI_{MUNIX}=\frac{\left(APB_{MUNIX}*FDI_{MUNIX}\right)}{ADM_{MUNIX}}$$

SI_MUNIX_ demonstrated high sensitivity (95%) and specificity (84%) and capable to detected SH even when CMAPs were unremarkable (Kim et al., 2016). SI_MUNIX_ appeared more encouraging than SI_CMAP_ in distinguishing ALS from cervical spondylotic amyotrophy (Zheng et al., 2019), but further confirmations are required.

All the above-mentioned neurophysiological parameters present, however, several limitations. The most important is the confounding contribute of aging to the intrinsic hand amyotrophy. In a recent study on patients without neuromuscular disorders, the authors found that APB, FDI, and ADM CMAPs decreased with age, approximately of 0.8/0.7/0.3 mV/year, respectively. Consistently, the SI decreased of about 0.15/year (Pechirra et al., 2022). Another potential confounding factor is the coexistence of median or ulnar entrapment neuropathies. Therefore, these indexes should be evaluated just in an extensive neurophysiological and clinical investigation (Corcia et al., 2021). Overall, SH seems a valuable tool in the diagnosis of ALS, but larger and longitudinal studies are required to identify a proper age-related neurophysiologic index (Lu et al., 2022).

## 3. Split-hand plus sign

In addition to the above mentioned observations, another intrinsic hand muscle seems to be relatively spared in ALS, the flexor pollicis longus (FPL). FLP although share the same innervation of APB from C8–T1 and median nerve, seems to be less involved in ALS (Menon et al., 2013a). This distinctive clinical feature has been called the “SH plus sign” (SHPS). However, the SHPS usefulness in clinical practice remains still poor characterized (Bae et al., 2014).

### 3.1. Pathophysiology

Although the mechanisms underlying SHPS in ALS remains to be elucidated, a cortical mechanism has been proposed. The thenar muscles play a critical role in fine movements, due to their wider cortical representation (Bae et al., 2013). Therefore, they are more susceptible than FPL to degeneration mediated by corticomotor neurons. Supporting this hypothesis is the significantly reduced cortical excitability from FPL rather than from APB, that gives evidence that FPL muscle receive a lower cortical output (Bae et al., 2013).

### 3.2. Neurophysiology

To date SHPS has been quantified only clinically, using the score at Medical Research Council (MRC) of APB divided by the score at MRC of FPL muscles, as median nerve strength index (MSI):


$$MSI=\frac{\left(APB_{MRC}\right)}{FPL_{MRC}}$$

A cut-off of 0.9 exhibited a good diagnostic accuracy (sensitivity: 85%; specificity: 86%), differentiating ALS from ALS mimic disorders. However, its preferential reduction in limb-onset ALS potentially limits its utility (Menon et al., 2013b). A subsequent study investigated the pathophysiology underlying SHPS. The authors calculated the corresponding NI as follows:


$$SHPI=\frac{\left(APB_{CMAP}\right)}{FPL_{CMAP}}$$

The SHPI was significantly reduced in ALS patients compared to controls. Interestingly, SHPI also demonstrated an inverse correlation with MEP amplitudes recorded over thenar muscle, supporting the cortical origin of SHPS in ALS (Bae et al., 2014). However, a clear cut-off of SHPI is still not available.

## 4. Split-elbow sign

Among the dissociated patterns of atrophy, Khalaf et al. (2019) proposed the “SE sign” (SES) to indicate the predominant weakness in ALS of biceps brachii (BB) compared to triceps brachii (TB) muscle. The SES is calculated as follows:


$$SES_{MRC}=\frac{\left(BB_{MRC}\right)}{TB_{MRC}}$$

Khalaf demonstrated that BB exhibited a smaller MRC score than TB muscle in a cohort of ALS patients (Khalaf et al., 2019). However, a subsequent study found opposite results, with a relative sparing of elbow flexion (Liu et al., 2021) with a predominant involvement of TB.

### 4.1. Pathophysiology

The SES further highlights the cortical hyperexcitability as potential mechanism underlying ALS, due to the wider cortical representation of BB than TB and its higher susceptibility to wasting and weakness in ALS (Khalaf et al., 2019; Pavey et al., 2021). Nonetheless, other pathophysiologic hypotheses, as the axonal hyperexcitability or the dysfunction of neuromuscular junction cannot be disclosed (Khalaf et al., 2019; Pavey et al., 2021).

### 4.2. Neurophysiology

The SES-related neurophysiologic index, the “SE index (SEI)” has been recently developed and is calculated, dividing the CMAP amplitude of BB by the CMAP of TB muscle, as follows:


$$SEI_{CMAP}=\frac{\left(BB_{CMAP}\right)}{TB_{CMAP}}$$

The SEI, with a cut-off of 0.65 (sensitivity of 71%; specificity of 61%) was significantly reduced in subjects with ALS compared to ALS mimicking disorders, regardless of the hand dominance. SEI may represent an early marker of disease, as it is more frequent in subjects with mild reduction in ALSFRS-R score. However, its usefulness in distinguishing ALS from mimic disorders is lower when the ALSFRS-R score is higher than 38. Larger studies may further contribute to define its utility in clinical settings and broaden ALS pathophysiology (Pavey et al., 2021).

## 5. Split-leg sign

In ALS, several studies reported an unbalanced muscular involvement in the lower-limbs during the course of the illness (Simon et al., 2015; Hu et al., 2019; Min et al., 2020). Simon et al. (2015) first found a significant asymmetrical wasting of plantar flexor (PF) compared to ankle dorsiflexion muscles (DF). Subsequent studies did not confirm such observation, but identified a preferential degeneration in extensor digitorum brevis (EDB) and tibialis anterior (TA) muscles, compared to abductor hallucis (AH) (Hu et al., 2019; Min et al., 2020), defining it as the “split-leg sign” (SL). Consistent with the SL sign, the foot-drop due to DF weakness is a common clinical manifestation and may be the prominent feature of lower-limbs involvement in ALS (Hu et al., 2019). On the other hand, several disorders may produce a foot-drop, including peroneal neuropathy at the fibular-head, lumbar spondylosis disease, and peripheral neuropathies (Stewart, 2008; Hu et al., 2019).

### 5.1. Pathophysiology

The mechanisms underlying the predominant involvement of EDB than AH are still not fully elucidated and a multifactorial origin has been assumed (Figure 2).

#### 5.1.1. Corticomotoneuronal dysfunction

The foot-drop represents a prominent feature of corticospinal tract injury. The greater density of the cortical motoneuronal projections to DF rather than PF muscles, may contribute to the pathogenesis of the SL (Brouwer and Ashby, 1992). Experimental studies evidenced higher cortical excitatory modulation in TA and higher inhibitory modulation in PF (Hudson et al., 2013). Although available human data are limited, TMS identified short-latency facilitation in motoneurons innervating TA and other small muscles of the foot, but rarely in soleus and gastrocnemius UMN (Hudson et al., 2013). Functional MRI during ankle DF/PF revealed a broader cortical representation for DF (Jiang et al., 2012). Thus, the corticomotoneuronal hyperexcitability is a key mechanism in degeneration of LMN involved in ankle dorsiflexors, that receive a greater innervation.

#### 5.1.2. LMN dysfunction

Lower motor neuron innervating muscles with more fast-twitching myofibers, larger soma and higher innervation ratios, such as TA and extensor digitorum longus, are especially prone to degeneration (Nijssen et al., 2017). Supporting this hypothesis are: the greater reduction of SL index (SLI) in patients with pure LMN diseases than in patients with ALS (Min et al., 2020); the predominant *F*-wave dysfunction in EDB compared to AH observed in patients with ALS. This finding could reflect a more severe hyperexcitability and neuronal loss among the EDB LMN, even in asymptomatic legs (Wang et al., 2019). Therefore, the LMN innervating the EDB may be involved in ALS earlier and deeper than those innervating the AH.

#### 5.1.3. The influence of physical activity

Although lower-limbs activity generally involves tonic activation of the glutei, vasti, and PF muscles during standing and walking, some studies observed a significant correlation between ALSFRS-R lower-limbs subscores and ankle DF strength rather than PF strength. The DF muscles may suffer from a greater oxidative stress (Liu et al., 2006). During the locomotion, the repeated phasic activation of DF might produce a greater oxidative stress in these antigravity muscles, leading to a premature dieback (Spiller et al., 2016). Further research should identify whether SL reflect a spinal mechanism or a down-stream process that secondarily involves LMN from cortical pathophysiology.

### 5.2. Neurophysiology

The SL has been quantified by several neurophysiological methods (Figure 2). Overall, the SLI corresponds to CMAP ratio of peroneal and tibial nerves (Simon et al., 2015; Hu et al., 2019; Min et al., 2020):


$$SLI_{CMAP}=PF_{CMAP}/DF_{CMAP}$$

Initially, the CMAP of peroneal and tibial nerves were, respectively recorded from TA and gastrocnemius. The higher mean SLI observed in ALS than controls suggested a dissociated involvement of lower-limbs with a greater reduction of PF_CMAP_ than DF_CMAP_ (Simon et al., 2015). However, due to the deepness of tibial nerve in the popliteal fossa, the nerve stimulation in this site may not excite all nerve fibers (Min et al., 2020). This technical issue produces an unusual variability of SLI, that collides with the common observation of foot-drop in patients with ALS (Hu et al., 2019; Min et al., 2020). Other authors therefore calculated the SLI recording peroneal and tibial nerve CMAPs from the belly of EDB and AH muscles (Hu et al., 2019; Min et al., 2020):


$$SLI_{CMAP}=EDB_{CMAP}/AH_{CMAP}$$

This SLI resulted lower in patients with ALS than in subjects with lumbar spondylosis, normal and neurological controls (Hu et al., 2019; Wang et al., 2019; Min et al., 2020), whereas no difference in SLI was observed between subjects with lumbar spondylosis, normal and disease controls (Min et al., 2020). The greater DF_CMAP_ reduction mainly occurs in ALS, whilst in lumbar spondylosis and in neurological controls, tibial and peroneal innervated muscles are affected simultaneously and symmetrically (Min et al., 2020). In a receiver operating characteristic (ROC) analysis, SLI (cut-off: 0.52) demonstrated a sensitivity of 80% and a specificity of 72% for ALS, resulting very useful for the differential diagnosis (Min et al., 2020). Indeed, in clinical practice lower limb-onset ALS can be easily misdiagnosed as lumbar spondylosis, especially for non-neuromuscular neurologists. In another study, SLI (cut-off: 0.61) failed in differentiating ALS from controls: sensitivity and specificity resulted of 67 and 54%, respectively (Wang et al., 2019). In a case/control study, the *F*-wave derived SLI (EDB/AH) demonstrated a higher sensitivity than the corresponding SLI_CMAP_ (Wang et al., 2019). Moreover, data about the clinical and prognostic implications of SLI are limited and conflicting. SLI inversely correlated with the duration of lower-limbs involvement (Hu et al., 2019), but did not correlate with muscle strength and functional outcomes (ALSFRS total score and lower-limbs sub-scores), suggesting that SLI does not link with lower-limbs disability (Menon and Vucic, 2014; Simon et al., 2015; Hu et al., 2019). Other studies observed instead a positive correlation between SLI and functional scales (Menon and Vucic, 2014; Corcia et al., 2021). The weak correlation between SLI and strength scores of the corresponding muscles could be due to the low sensitivity of the manual strength tests (Menon and Vucic, 2014), as for SI. Based on all the above-mentioned observations, the usefulness of SLI for an earlier diagnosis of ALS and its clinical correlations need to be further elucidated.

## 6. Discussion

This review indicates that the dissociated wasting of intrinsic hand and foot muscles is common in ALS. SH and SL represent typical manifestations of ALS, particularly in the earliest stages, when clinical signs are focal. The highest amount of data belongs to the SH, while the SL (as well as SE and SHP signs) is actually poorly characterized. Although several NIces have tried to assess the SH, the SI_CMAP_ represents the most reliable marker and may help to differentiate ALS from mimic syndromes (Hu et al., 2019; Corcia et al., 2021).

On the other hand, the diagnostic usefulness of SLI_CMAP_ still remains debated (Simon et al., 2015; Liu et al., 2021; Wang et al., 2019; Min et al., 2020). In the largest case-control studies SLI was calculated by recording CMAPs from EDB and AH muscles, resulting useful for the differential diagnosis of ALS in most, but not all the studies (Simon et al., 2015; Hu et al., 2019; Wang et al., 2019; Min et al., 2020). The application of SLI in the clinical practice could be relevant, as the lower-limbs ALS can be misdiagnosed as lumbar spondylosis. Several observations suggested also a prognostic role for SI, rather than SLI, as it correlates with disease progression and prognosis (Corcia et al., 2021). Finally, just preliminary observations on SE and SHPS are available and no firm conclusion can be actually drawn.

The diagnostic role of CMAP driven SI and SLI is supported by several studies and do not need a sophisticated neurophysiological equipment. Other neurophysiological methods, although potentially more sensitive, requires additional software (e.g., MUNIX) or are less likely to be performed in the routine neurophysiological assessment (*F*-waves persistence driven). Nevertheless, some possible confounding factors, such as the mononeuropathies of median nerve at wrist and the peroneal neuropathy at the fibular head, should be addressed in the evaluation of SI and SLI. Normative values of SI and SLI are also uncertain, as available data are extremely variable and the contribute of aging is still unclear (Corcia et al., 2021; Pechirra et al., 2022). Finally, the underlying mechanisms of SLI and SLI are also undetermined, and current evidences indicate an involvement of both cortical and spinal/peripheral pathways. The higher use in humans of APB, FDI, TA, and EDB muscles, compared to ADM, AH, gastrocnemius, leads to a greater oxidative stress and metabolic demand for both UMN and LMN innervating such muscles. This could explain the typical topography of intrinsic hand and foot muscle hypotrophies and the presence of SH in MND without UMN involvement, as Kennedy’s disease. Overall, SI and SLI are reliable neurophysiological markers. Although diagnostic cut-offs are uncertain, their diffuse use in clinical settings may provide new insight into the pathophysiology of ALS. Further studies are, however, needed to clarify their pathophysiology and clinical correlations.

## Author contributions

All authors listed have made a substantial, direct, and intellectual contribution to the work, and approved it for publication.
